# Water-richness evaluation method and application of clastic rock aquifer in mining seam roof

**DOI:** 10.1038/s41598-024-57033-x

**Published:** 2024-03-18

**Authors:** Mei Qiu, Zhendong Shao, Weiqiang Zhang, Yan Zheng, Xinyu Yin, Guichao Gai, Zhaodi Han, Jianfei Zhao

**Affiliations:** 1 College of Earth Sciences and Engineering, Shandong University of Science and Technology, Qingdao, 266590 China; 2 Key Laboratory of Sedimentary Mineralization and Sedimentary Minerals in Shandong Province, Shandong University of Science and Technology, Qingdao, 266590 China; 3 Shandong Shengyuan Geological Exploration Co., Ltd, Taian, 271000 China; 4 Jinan Rail Transit Group CO., LTD, Jinan, 250013 China

**Keywords:** Water-richness, Clastic rock, Neural network, Combination weighting method, Unascertained measures theory, Hydrology, Natural hazards

## Abstract

Clastic rock aquifer of the coal seam roof often constitutes the direct water-filling aquifer of the coal seam and its water-richness is closely related to the risk of roof water inrush. Therefore, the evaluation of the water-richness of clastic rock aquifer is the basic work of coal seam roof water disaster prevention. This article took the 4th coal seam in Huafeng mine field as an example. It combined the empirical formula method and generalized regression neural network (GRNN) to calculate the development height of water-conducting fracture zone, determined the vertical spatial range of water-richness evaluation. Depth of the sandstone floor, brittle rock ratio, lithological structure index, fault strength index, and fault intersections and endpoints density were selected as the main controlling factors. A combination weighting method based on the analytic hierarchy process (AHP), rough set theory (RS), and minimum deviation method (MD) was proposed to determine the weight of the main controlling factors. Introduced the theory of unascertained measures and confidence recognition criteria to construct an evaluation model for the water-richness of clastic rock aquifers, the study area was divided into three zones: relatively weak water-richness zones, medium water-richness zones, and relatively strong water-richness zones. By comparing with the water inrush points and the water inflow of workfaces, the evaluation model's water yield zoning was consistent with the actual situation, and the prediction effect was good. This provided a new idea for the evaluation of the water-richness of the clastic rock aquifer on the roof of the mining coal seam.

## Introduction

In the process of coal mine production, mine water disasters are major threats, and a common type of them is the water disaster to the coal seam roof aquifer. In various eras of coal fields in China, clastic rock aquifers such as sandstone and conglomerate are widely developed in coal-bearing strata^1^. During coal mining, cracks and fractures occur in the roof strata. Once these cracks and fractures communicate with each other, they form a water channel, guiding water from the roof aquifer into the mining site, causing instantaneous large-scale water inrush disasters. In the mild case, it can cause mining machinery losses, and in the severe case, it can lead to flooding of wells or even catastrophic casualties. The clastic rock aquifer within the range of the water-conducting fracture zone of the coal seam roof becomes the direct water-filling aquifer of the mining coal seam, and its water-richness directly determines the occurrence and inflow of water from the roof. Therefore, evaluating the water-richness of coal seam roof clastic rocks has practical guiding significance for the safety production of mines.

At present, the methods for evaluating the water-richness of coal seam roof clastic rock aquifers in China are mainly divided into three categories^2,3^: Firstly, based on the data of unit water inflow, the classification of water-richness is directly based on the "Detailed Rules for Coal Mine Water Prevention and Control"^4^. This method is the most accurate, but it needs to be based on a large amount of hydrological borehole data, and during the mining stage, the boreholes for pumping tests are often scarce and unevenly distributed^5^. The second is to use geophysical methods such as transient electromagnetic method, high-resolution direct current method, audio frequency electric perspective method, etc^6,7^, however, geophysical methods are often costly, and the multiplicity of geophysical results cannot be avoided^8^. The third is the comprehensive analysis method of multiple factors. This method involves in-depth exploration of hydrogeological exploration data, selecting and weighting factors affecting water-richness, and constructing a water-richness evaluation model based on statistical or fuzzy mathematical methods, it comprehensively considers the weights of various influencing factors and indicators, and the zoning results obtained can truly reflect the characteristics of aquifer water-richness^9^. Currently, it is the most widely used method, but the degree of hydrogeological exploration in most coal mining areas in China is relatively low, and the unit water inflow data of aquifers is limited, which cannot fully reflect the distribution characteristics of aquifers. In addition, some scholars had a relatively single method for weighting impact indicators, which affects the accuracy of water-richness evaluation^10^.

Through comprehensive comparison of the above-mentioned methods for evaluating the water-richness of the coal seam roof, this study decided to use the multi-factor comprehensive analysis method. The main idea of the multi-factor comprehensive analysis method is to first establish an index system for the indicators influencing water-richness, and then couple the indicators with their weights to establish an evaluation model. Yu et al.^11^ determined the weights of aquifer influencing factors using Analytical Hierarchy Process (AHP) and conducted water-richness zoning using SURFER software. Tang et al.^12^ combined AHP with entropy weight method and established an evaluation model using ArcGIS software, which yielded reliable results. Bi et al.^13^ combined AHP with independent weight coefficient method to establish an evaluation model with high accuracy. Wang et al.^14^ also used AHP to establish an evaluation system and conducted water-richness zoning in the study area, providing scientific guidance for the prevention and control of water disasters in mining areas. Qiu et al.^15^ successfully applied Fuzzy Delphi Analytic Hierarchy Process (FDAHP) combined with entropy weight method, introducing the Technique for Order Preference by Similarity to an Ideal Solution (TOPSIS) method to construct an aquifer richness evaluation model. In another study area, Qiu et al.^5^ combined FDAHP with grey correlation analysis to establish a water-richness zoning model with high accuracy. Huang et al.^10^ combined FDAHP with entropy weight method to establish a water-richness zoning evaluation model through the theory of unascertained measures, and the model has certain reliability. Li et al.^16^ and Gong et al.^17^ applied Back-Propagation neural network (BP) in aquifer richness evaluation, summarizing the distribution pattern of aquifer richness and achieving accurate prediction. Li et al.^18^ used random forest to establish a model and evaluate the weights of various influencing indicators, conducting zoning of water-richness in the study area, with results meeting the accuracy requirements. Some scholars only used one weighting method, and the obtained weights may be too subjective or objective; Some scholars had also adopted various weighting methods but only used simple geometric averaging to combine weights, lacking scientific rigor. So how to balance the influence of subjective and objective weights and improve the accuracy of weights has become an urgent problem to be solved.

This article took the 4th coal seam in Huafeng mine field as the research object. Firstly, the Generalized Regression Neural Network (GRNN) and the "three down" regulation formula were comprehensively used to calculate the development height of the water-conducting fracture zone in the coal seam roof, to more accurately determine the vertical spatial range of water-richness evaluation. On this basis, the analysis was conducted from two aspects: lithological structural characteristics and structural development characteristics. Lithological structural characteristics refer to the intrinsic properties of the rock itself, including its composition and internal structure, manifested as rock type, rock structure, thickness of sandstone layer, etc. Structural development characteristics refer to the changes in geological structure and geomorphological formations caused by geodynamic effects, manifested as the development of folds and faults formations. The depth of the sandstone floor, brittle rock ratio, lithological structural index, fault strength index, fault intersections and endpoints density were selected as the main controlling factors to evaluate the water-richness of the clastic rock aquifer in the study area. Based on the minimum deviation method, the main controlling factors' subjective and objective weights obtained from Analytic Hierarchy Process (AHP) and Conditional Entropy Improved Rough Set Theory were effectively fused to obtain comprehensive weights, making the weight results more reasonable. Introducing the theory of unascertained measurement and confidence criteria, a water-richness evaluation model for the clastic rock aquifer of the 4th coal seam roof in Huafeng Coalfield was constructed. Compared with the water inrush points and the water inflow of some workfaces in the mine, the zoning prediction results of the evaluation model were relatively consistent. The method in this article provided a new approach for evaluating the water-richness of clastic rock aquifers in coal seam roof during mining.

## Study area

### Overview of geological structure

**Figure 1:**
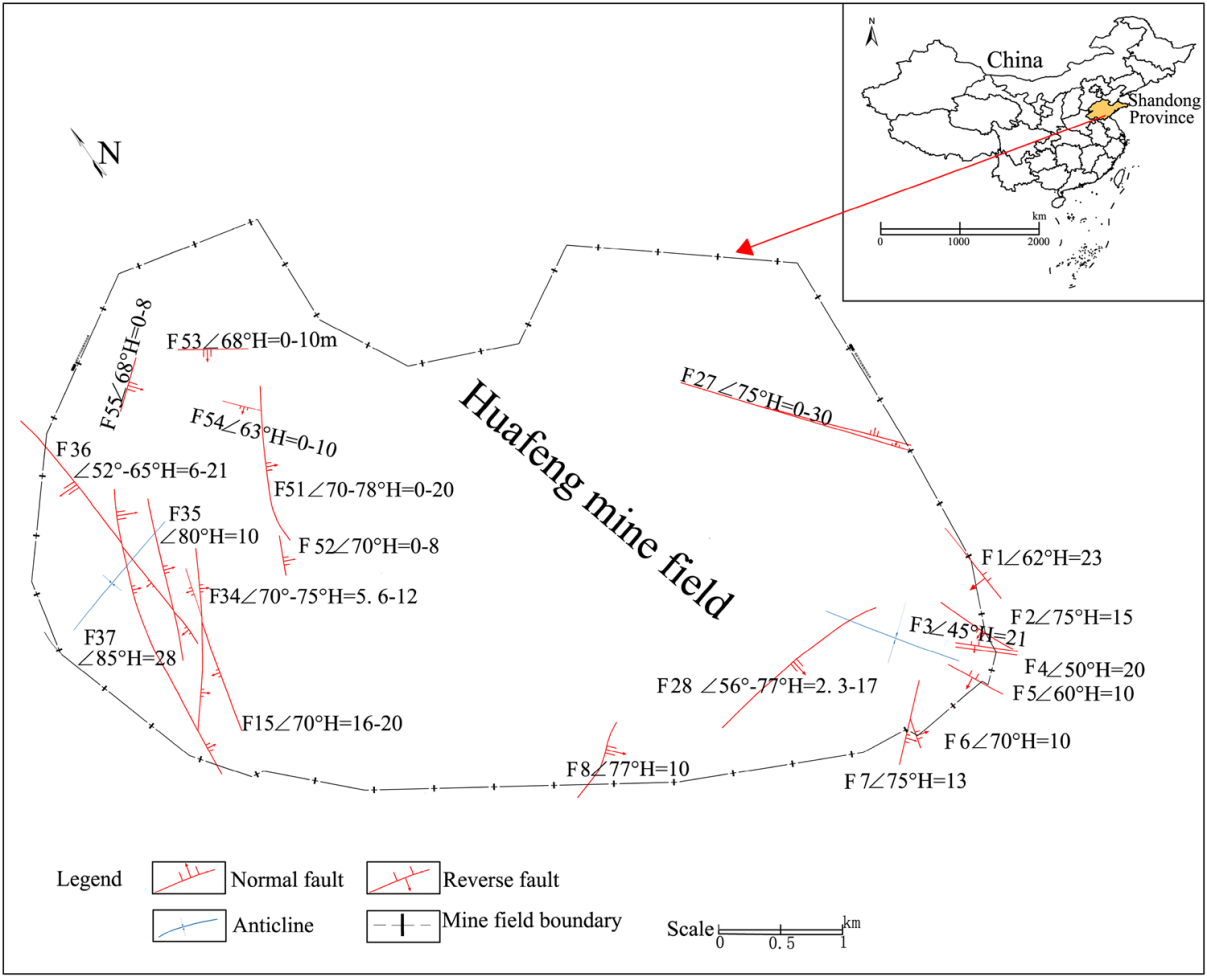
Structural outline map of Huafeng mine field.

Huafeng mine field lies in Huafeng Town, Ningyang County, Shandong Province, China. The site lies between 117° 07′ 28″ E and 117° 11′ 11″ E longitude and 35° 51′ 49″ N and 35° 54′ 52″ N latitude. The Huafeng mine field is a fault block depression bounded by faults on three sides, namely the east, north, and west. It is generally a dustpan shaped syncline structure that plunges towards the northeast, with a dip angle of 17° to 40°. The structure of the mine field is relatively simple (Fig. 1). Local normal faults with a drop of no more than 30 m and a few reverse faults have developed in the mine field, which generally extend relatively short and tip out towards the deep. In addition, multiple secondary folds have also developed in the mine field. However, except for the two obvious synclines at the turning points of the two wings, the scale is generally very small, and the deformation towards the deep is unusually gentle and even disappears. The strata in the study area include the Middle Lower Ordovician, Carboniferous Yuemengou Group Benxi Formation, Carboniferous Permian Yuemengou Group Taiyuan Formation, Permian Yuemengou Group Shanxi Formation, Permian Shihezi Formation, Paleogene Lower Guanzhuang Group, and Quaternary System. The main coal bearing strata have a total thickness of 325.86 m and a total of 24 coal layers. The Shanxi and Taiyuan formations are the main coal bearing strata.

### Hydrogeological overview

The aquifer in the Huafeng mine field consists of Quaternary aquifer sand and gravel layer, Paleogene conglomerate, Shanxi Formation sandstone, Taiyuan Formation thin layer limestone, Xucao limestone, and Ordovician limestone. Each aquifer forms a multi-layer structure groundwater type in the coal field, belonging to the northern type of multi aquifer karst fissure water filled deposit. The main aquifers are the Paleogene reddish brown clayey siltstone beneath the conglomerate, the Shihezi Formation variegated clay rock, and the siltstone, mudstone, and clay rock between the various aquifers in the coal bearing strata.

**Figure 2:**
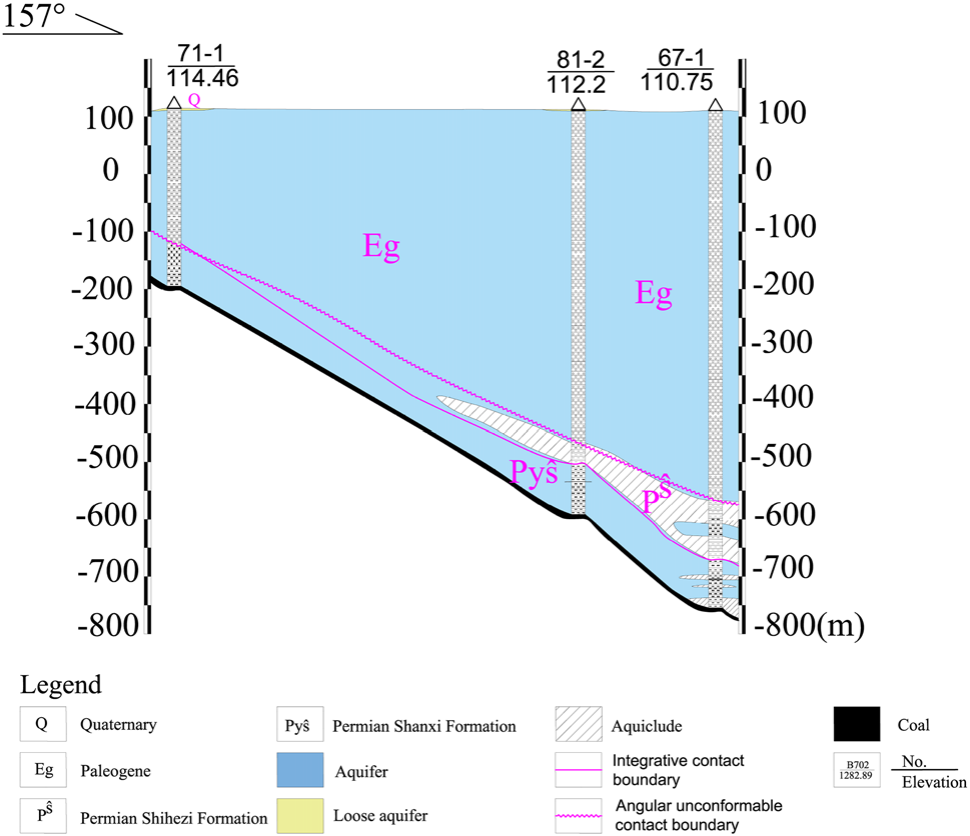
Hydrogeologic profile of 4th coal seam roofs.

The overlying aquifer of 4th coal seam in the mine field is composed of Quaternary water-bearing gravel layer, Paleogene conglomerate, and Shanxi Formation sandstone. The Paleogene conglomerate aquifer directly covers the coal bearing strata and has a small distance between the shallow part and the 4th coal seam, which has a significant impact on the mining of the 4th coal seam; The sandstone of the Shanxi Formation mainly refers to the sandstone on the top and bottom of the 4th coal seam, which under normal circumstances mainly leaks into the mine in the form of water pouring (Fig. 2).

## Data and method

**Figure 3:**
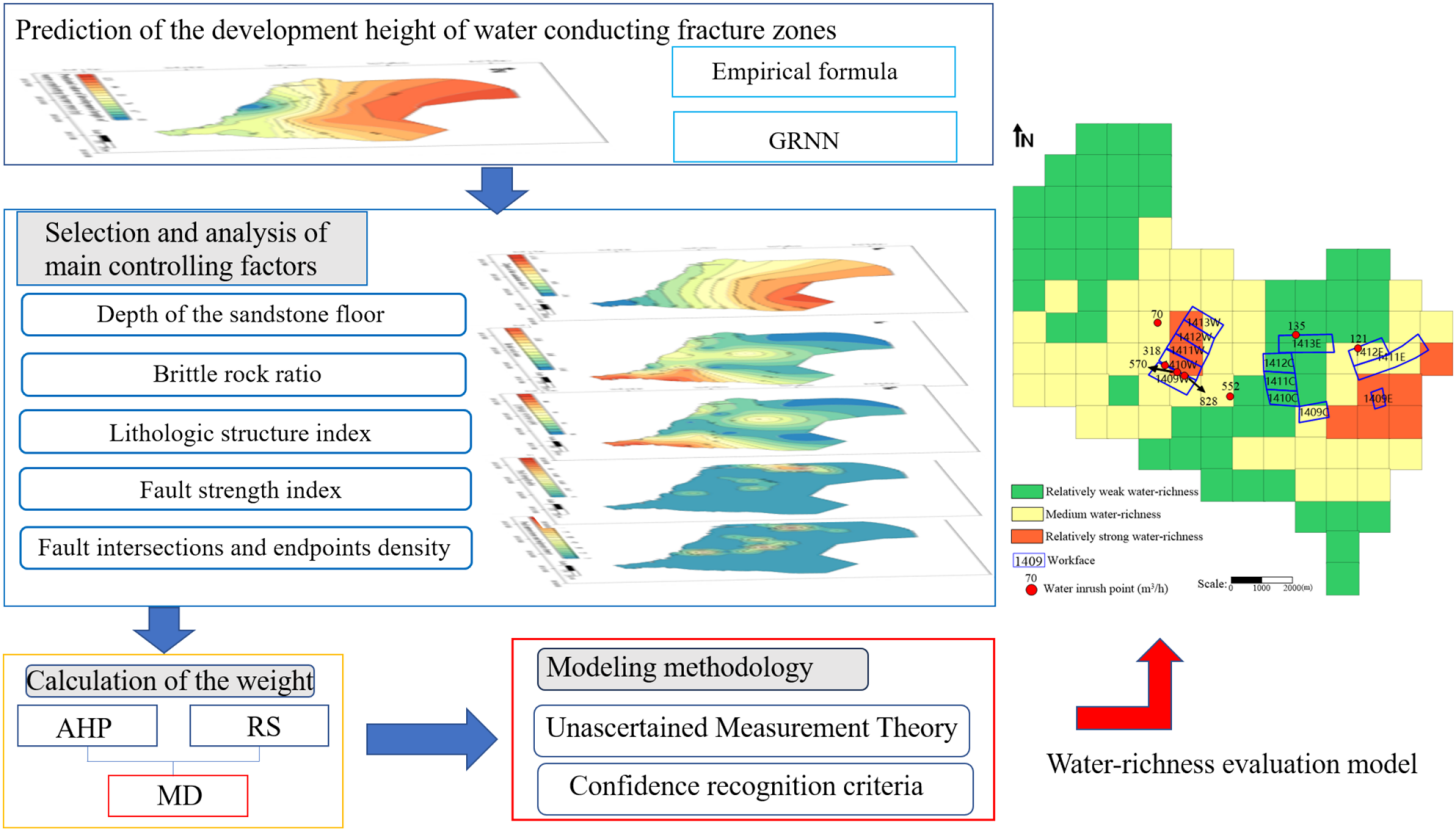
Technology roadmap.

The evaluation of the water-richness of the clastic rock aquifer on the roof of the mining coal seam mainly included the following steps: (1) By using empirical formulas and GRNN neural networks, the height of the development of water-conducting fracture zones above coal seams was calculated. (2) Based on the multi-factor control mechanism of water-richness in the coal seam roof and hydrogeological data of the study area, the main controlling factors of the water-richness in the clastic rock aquifer above the mining coal seam were selected. (3) The AHP method and the rough set theory improved by conditional entropy were used to calculate the subjective and objective weights of each main controlling factor, and comprehensive weight was obtained based on the minimum deviation. (4) The evaluation model of water-richness was established for the study area using the theory of uncertain measurement and confidence criteria. The water-richness of clastic rock aquifer above the coal seam was analyzed, and a grading prediction was made (Fig. 3).

### Prediction of the development height of water-conducting fracture zones

**Figure 4:**
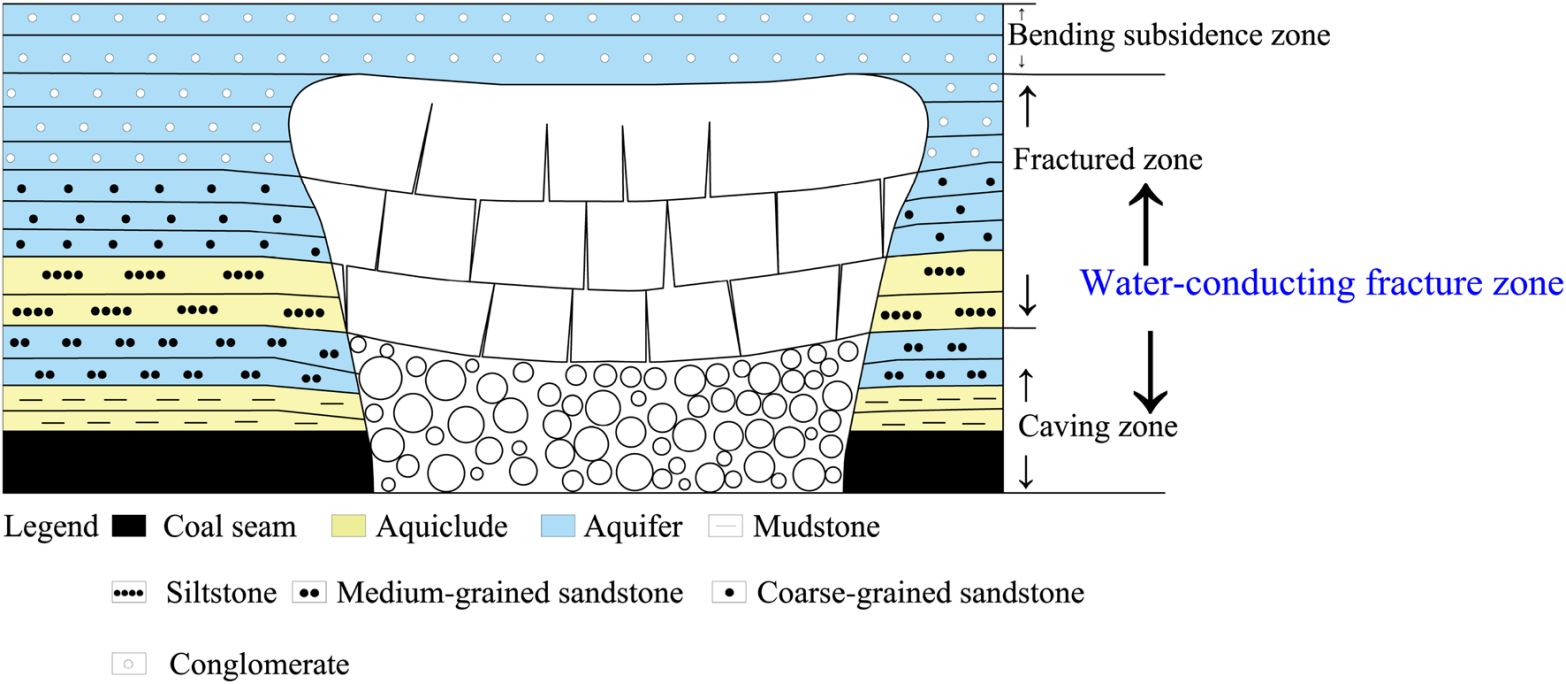
Schematic diagram of the "upper three belts".

After coal seam mining, the overlying rock above the goaf undergoes damage and deformation. According to the "upper three zones" theory of coal mining, the areas of damage and deformation are divided into caving zone, fractured zone, and bending subsidence zone. Among them, the caving zone and fractured zone are collectively referred to as water-conducting fracture zone. (Fig. 4) The water-conducting fracture zone connects the clastic rock aquifer on the coal seam roof with the workface. The clastic rock aquifer within this height range constitutes the direct water-filling aquifer for water inrush on the coal seam roof, and the water-conducting fracture zone becomes the main channel for water damage on the coal seam roof. It is of great significance for the safe mining of coal seams to partition and evaluate the water-richness of the coal seam roof directly filled aquifer within the height range of the water-conducting fracture zone during the coal seam mining process.

### Empirical formula method

At present, on-site technical personnel in coal mines in China widely use the formula provided in the "Regulations on the Retaining and Mining of Coal Pillars in Buildings, Water Bodies, Railways, and Main Tunnels"^19^ to calculate the development height of water-conducting fracture zones. In the regulations, based on the size of uniaxial compressive strength, the roof lithology is divided into four types: hard, medium-hard, weak, and extremely weak, and different formulas are used according to the degree of hardness^20^.

The calculation formula for the development height of water-conducting fracture zones with different lithology is as follows:

1 $${\text{Hard}}:H = \frac{100\sum M }{{1.2\sum M + 2.0}} \pm 8.9,$$

2 $${\text{Medium}} - {\text{hard}}:H = \frac{100\sum M }{{1.6\sum M + 3.6}} \pm 5.6,$$

3 $${\text{Weak}}:H = \frac{100\sum M }{{3.1\sum M + 5.0}} \pm 4.0,$$

4 $${\text{Extremely weak}}:H = \frac{100\sum M }{{5.0\sum M + 8.0}} \pm 3.0,$$

where∑*M* is the total thickness of coal seam mining, in meters, and *H* is the height of water-conducting fracture zone, in meters.

The empirical formula method is simple and fast, but only considers the strength of coal seam overlying rock and mining thickness, and the specific geological and mining conditions of different workfaces are not the same. Therefore, the predicted values obtained by this method are only for reference and need to be further analyzed in conjunction with other methods.

### Neural network method

With the development of computer technology, neural networks have been widely used for predicting the development height of water-conducting fracture zones. Its advantage is that it can process a large amount of data, extract useful features from it, and better identify trends related to the height of hydraulic fracture zones. A trained neural network model can be used for online prediction in actual production processes, which means it can predict real-time data, assist in practical applications, and adjust and optimize accordingly.

Generalized regression neural network (GRNN) is a type of Radial Basis Function Neural Network (RBF) that has been widely used in the field of regression prediction. It has stronger approximation ability and learning speed compared to RBF networks, strong nonlinear mapping ability, and learning speed, and simple structure with single parameter setting^21,22^.

**Figure 5:**
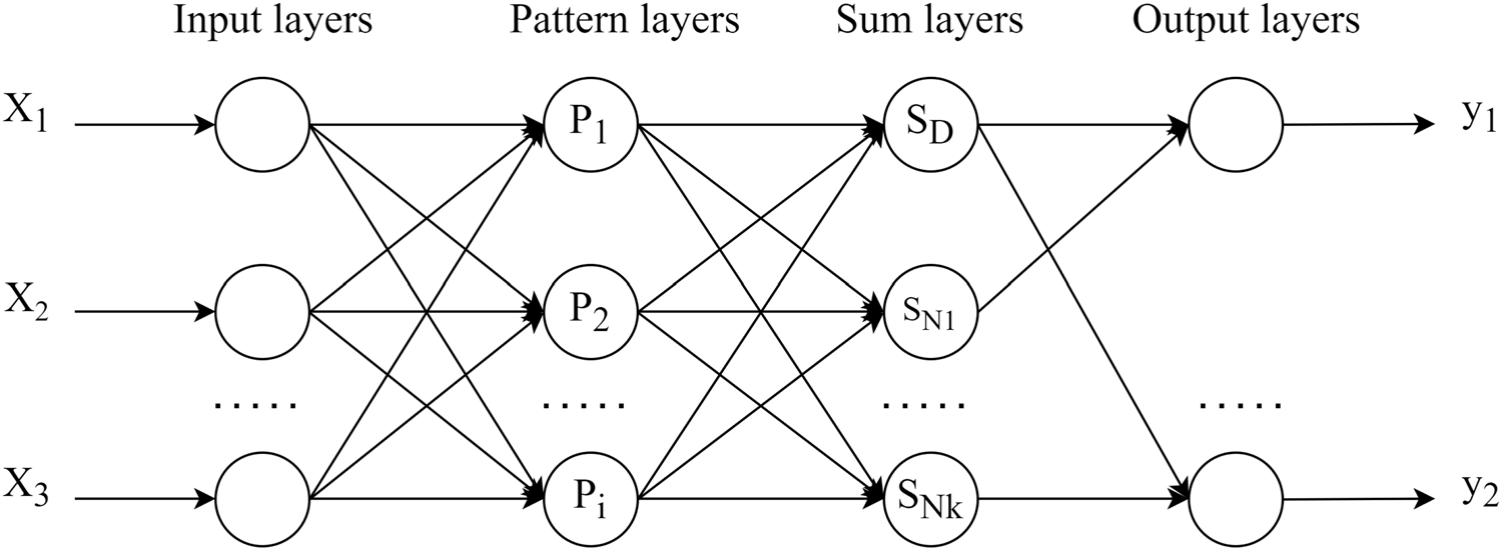
Structure of GRNN neural network model.

The structure of GRNN neural network is shown in Fig. 5, including input layer, pattern layer, summation layer, and output layer^23–26^.

1. Input layers: the number of input neurons is equal to the dimension of the input vector in the training sample, and the input neurons directly enter the next pattern layer.
2. Pattern layers: the number of neurons in the pattern layer is equal to the number of neurons in the input layer, and non-linear transformation is performed on the output from the input layer. The transfer function of the $i$ neuron is often used as follows:
  5 $$P_{i} = exp[ - \frac{{(X - X_{i} )^{T} (X - X_{i} )}}{{2\sigma^{2} }}] \, i = 1,2, \cdot \cdot \cdot ,n$$
  where *P*_*i*_ is output layer neuron model; *X* is input vector for the network; *X*_*i*_ is a learning sample for the corresponding *i* neuron; σ is the smoothing factor; *n* is the number of training samples.
3. Sum layers: this layer is used to calculate the total output from the pattern layer, and there are two types of neurons applied to it.

The first neuron algorithm adds all neurons in the pattern layer, with a connection weight of 1 between each neuron in the pattern layer and the transfer function is:

6 $$S_{D} = \sum\limits_{i = 1}^{{\text{n}}} {p_{i} = } \sum\limits_{i = 1}^{n} {\exp \left( { - \frac{{\left( {X - X_{i} } \right)^{T} \left( {X - X_{i} } \right)}}{{2\sigma^{2} }}} \right)}$$

Another calculation formula has added weighted sum, where the *j* element in the *i* output sample *Y*_*i*_ is the connection weight between the *i* neuron in the pattern layer and the *j* element in the summation layer and the neuron. Its transfer function is:

7 $$S_{{N_{j} }} = \sum\limits_{i = 1}^{n} {Y_{ij} p_{j} } = \sum\limits_{i = 1}^{n} {Y_{i} \exp \left( { - \frac{{\left( {X - X_{i} } \right)^{T} \left( {X - X_{i} } \right)}}{{2\sigma^{2} }}} \right)}$$

where *Y*_*i*_ is the *i* output sample; *n* is the number of nodes in the pattern layer; *k* is the dimension of output vector; *Y*_*ij*_ is the *j* value of the result vector in the *i* training sample.

$S_{D}$ as the denominator of the output layer, this function is mainly used for normalization to ensure the stability of the output results, its calculation is relatively simple, and can avoid the numerical instability caused by the molecular part of the value is too large. $S_{{N_{j} }}$ as the molecule of the output layer, by weighting the contribution of each sample, this function is able to more accurately reflect the similarity between the input vectors and the training samples, thus improving the accuracy of the prediction.

(4) Output layers: the number of neurons in the output layer is equal to the dimension *k* of the output vector in the training sample, and the output result of each neuron is:
  8 $$y_{i} = \frac{{S_{{N_{j} }} }}{{S_{D} }} \, j = 1,2, \cdot \cdot \cdot ,k$$

where *y*_*i*_ is the output of the *j* node in the output layer, which is the predicted result.

The GRNN neural network will be trained by the measured cases of the development of water-conducting fracture zones with similar geological conditions to the study area. Suitable influencing factors need to be selected as input values for the input layer. Based on the drilling data and previous studies in the research area, four factors, namely coal seam thickness, proportion coefficient of hard rock lithology, dip length of the workface, and mining depth, were selected as the influencing factors for the development height of the water-conducting fractured zone.

- Coal seam thickness (*m*). This factor plays a crucial role in determining the development height and serves as the primary controlling factor for determining the conduit height in traditional empirical formulas. With increasing coal seam thickness, the caving zone expands, leading to a corresponding increase in the development of the water-conducting fractured zone.
- Proportion coefficient of hard rock lithology (*b*)^15^. This factor can replace the two influencing factors of the uniaxial compressive strength and structural type of the roof combination rock layer, reflecting the strength type and lithology combination of the coal seam roof.
- Dip length of the workface (*l*). Prior to the full exploitation of coal seams, the dip length of the workface has a significant impact on the development of the water-conducting fractured zone, with the development height increasing as the workface advances. After the coal seam has been fully exploited, the effect of the dip length of the workface on the development of the fractured zone is not significant.
- Mining depth (*s*). As the mining depth increases, the mine pressure also increases, causing previously unconnected fractures in the overlying strata of the coal seam to become interconnected, thus forming water-conducting channels. Therefore, mining depth can be considered as a influencing factor.

After the training of the GRNN neural network was completed by the measured data, the above influencing factors were substituted into the network as the input layer data, and the predicted value of the height of the development of the water-conducting fracture zone in the roof of the coal seam in the study area could be obtained.

### Analysis of the main controlling factors of water-richness

The selection of the main controlling factors of the coal seam roof aquifer is the prerequisite and foundation for the evaluation of water-richness. Reasonable selection of the main controlling factors can greatly improve the scientific and reliable evaluation of the water-richness of the coal seam roof aquifer. Based on the hydrogeological data of the study area, this article selected the depth of the sandstone floor, brittle rock ratio, lithological structure index, fault strength index, fault intersections and endpoints density as the main controlling factors for evaluating the water-richness of the clastic rock aquifer on the roof of the 4th coal seam in Huafeng mine field.

- Depth of the sandstone floor (*D*), is the bottom burial depth of the sandstone in the upper part of the mining coal seam. The main impact of this factor is that as the depth increases, the static pressure of the rock will also gradually increase, and the degree of compaction of the sandstone will also increase. This will reduce the probability of cracks in the rock layer and reduce the water-richness^27^.
- Brittle rock ratio (*R*), is the ratio of brittle rock thickness to plastic rock thickness within the statistical range. National and international scholars have evaluated the brittleness of rocks from different perspectives, such as mineral composition^28^, stress–strain curves based on rock brittleness characteristics^29^, and rock mechanics parameters^30^. In this study, the brittleness of rocks is analyzed based on the rock mechanics parameters and mineral composition of each stratum in the coal seam roof.

From the analysis of rock mechanics parameters, combined with the preliminary geological survey report of the study area, the brittleness of rocks is quantified by calculating the area enclosed by the uniaxial tensile-uniaxial compressive strength curve^28^. The formula is shown below:

9 $$B{ = }\sigma_{c} \sigma_{t} /2,$$

where *B* is the brittleness of the rock, $\sigma_{c}$ is the uniaxial compressive strength of the rock, $\sigma_{t}$ is the uniaxial tensile strength of the rock. The rock mechanics parameters and brittleness degree of the roof strata of the 4th coal seam in Huafeng coal mine are shown in Table 1.

**Table 1:** Rock brittleness evaluation table.

Rock layer	Tensile strength/MPa	Compressive strength/MPa	*B*
Conglomerate	3.2	68.75	110.00
Coarse-grained sandstone	3.1	66.2	102.61
Medium-grained sandstone	3.7	65.19	120.60
Fine-grained sandstone	7	73.6	257.60
Siltstone	2.3	39	44.85
Mudstone	0.88	11.7	5.15

In terms of rock mineral composition, conglomerate, coarse-grained sandstone, medium-grained sandstone, and fine-grained sandstone in the strata often contain brittle minerals such as quartz, while siltstone and mudstone commonly contain clay minerals.

Taking all factors into consideration, we classified fine-grained sandstone, medium-grained sandstone, coarse-grained sandstone, and conglomerate as brittle rocks, while classifying siltstone and mudstone as plastic rocks.

Compared to plastic rocks, brittle rocks are more prone to generating a large number of cracks under stress, greatly enhancing their water permeability and storage capacity. Therefore, the brittle rock ratio can be used as a factor affecting the water-richness of aquifers, and the larger the ratio, the stronger the water-richness of aquifers^11^. The formula for calculating the brittle rock ratio is as follows:

10 $$R = \left( {a + b + c} \right)/d,$$

where *R* is the brittle rock ratio; *a* is the thickness of conglomerate and coarse-grained sandstone, in meters; *b*, *c* is the thickness of medium-grained sandstone and fine-grained sandstone, in meters; *d* is the thickness of plastic rocks such as siltstone and mudstone, in meters.

c. Lithological structure index (*L*). Using this factor to reflect the lithology, thickness, and combination characteristics of sand and mudstone within the range of water-conducting fracture zones, the larger the lithological structure index, the better the water-richness of the aquifer. The calculation method of lithological structure index in this paper is as follows: The thickness of fine-grained sandstone, medium-grained sandstone, conglomerate, etc., is multiplied by an equivalent coefficient to convert it into the thickness of coarse-grained sandstone, and then multiplied by the structural coefficient^31^. The structural coefficient refers to the coefficient determined by the sand-mud combination structure of the rock layer. The structural coefficients are taken as shown in Table 2.

The formula for calculating the lithological structure index is as follows:

11 $$L = (a \times 1 + b \times 0.8 + c \times 0.6) \times g,$$

where *L* is the lithological structure index; *a* is the thickness of conglomerate and coarse-grained sandstone, in meters; *b, c* is the thickness of medium-grained sandstone and fine-grained sandstone, in meters; *g* is the structural coefficient.

**Table 2:** Structural coefficients of the lithological structure index.

Thickness ratio of sandstone within the extent of the water-conducting fracture zone (%)	Structural coefficient
> 80	1
55 ~ 80	0.8
45 ~ 55	0.6
20 ~ 45	0.4
≤ 20	0.2

d. Fault strength index (*I*). Faults provide storage space and migration channels for groundwater, connecting coal seams and roof aquifers, and are important factors causing water inrush from coal seam roof aquifers. The fault strength index, which combines the fault drop, horizontal extension length, and number of faults, can quantitatively evaluate the development of faults and objectively and truly reflect the complexity of faults. The formula is^32^:
  12 $$I = \frac{{\sum\limits_{i = 1}^{n} {H_{i} L_{i} } }}{S},$$
  where *I* is the fault strength index; *n* is the total number of faults per grid; *H*_*i*_ is the drop of the *I* fault in a certain grid, in meters; *L*_*i*_ is the length of the *I* fault in a certain grid, in meters; *i* = 1, 2, …, *n*; *S* is grid area, in square meters.
e. Fault intersections and endpoints density (*F*). At the intersections and endpoints of faults, due to stress concentration and the cutting effect between faults, the degree of rock fracture is greater and the cracks are more developed, increasing the possibility of water inrush from the coal seam floor. Fault intersections and endpoints density is the sum of fault intersections and pinch points per unit area divided by the area of the area, which can intuitively reflect the complexity of the fault, the formula is as follows:
  13 $$F = \frac{n}{S},$$
  where *F* is the fault intersections and endpoints density; *n* is the total number of intersections and endpoints of all faults in the grid; *S* is grid area, in square meters.

**Figure 6:**
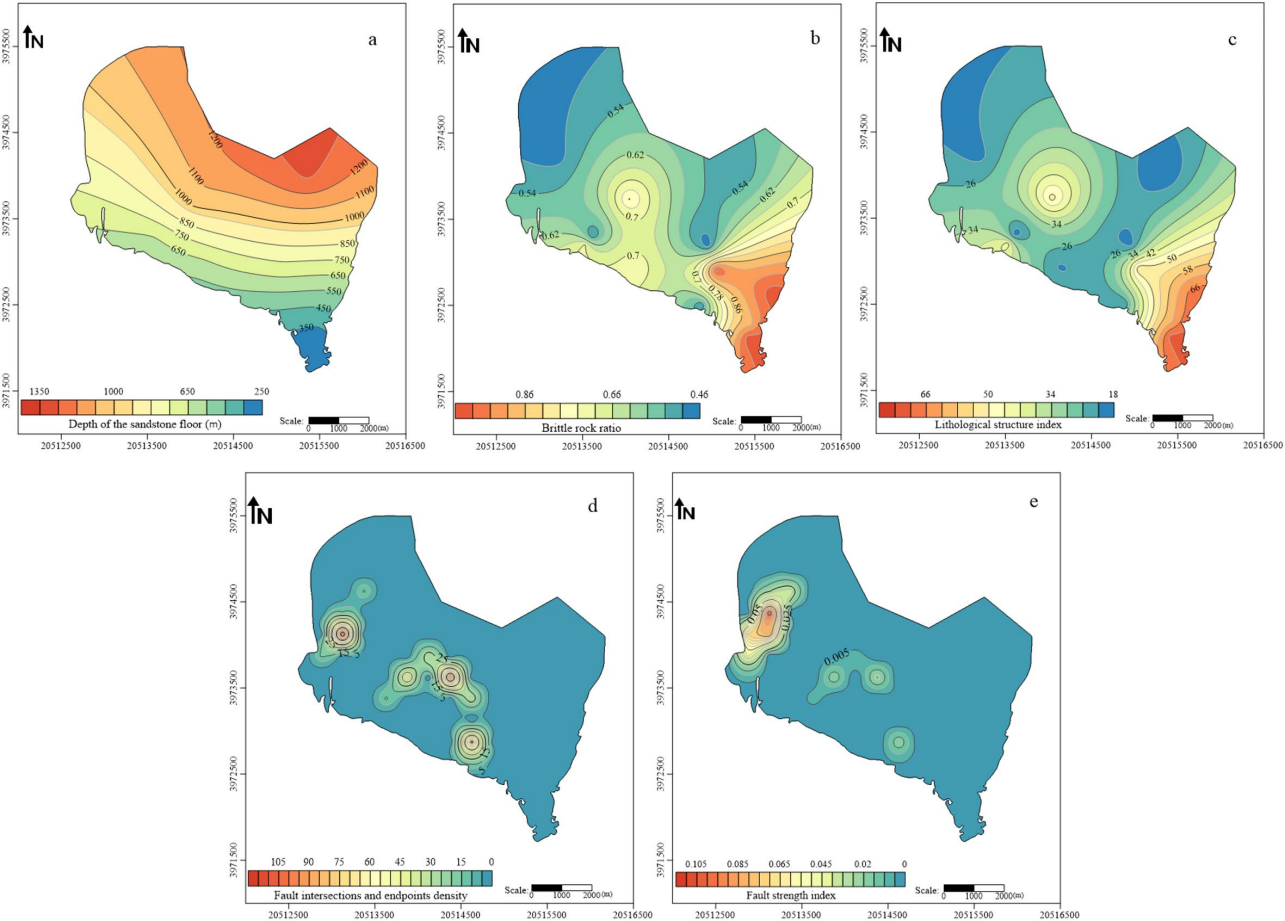
The main controlling factors of water-richness evaluation.

By conducting statistics and analysis on the existing drilling data and geological data exposed in the Huafeng mine field, the Golden Software Surfer software was used to draw thematic maps of various main control factors, as shown in Fig. 6:

### Calculation of weight of main controlling factors

The weight of the main controlling factors plays a crucial role in accurately determining the evaluation results when conducting a multi-factor coupling evaluation of the water-richness of the coal seam roof aquifer. In traditional methods, weighting methods are too single, subjective or objective, or only use simple geometric averaging methods, which do not effectively combine subjective and objective weights, affecting the accuracy of weights. This article used the rough set theory method improved by conditional entropy and AHP to calculate subjective and objective weights respectively, and then effectively combined subjective and objective weights based on the minimum deviation method.

### Calculating subjective weights based on AHP

Analytic Hierarchy Process (AHP) is a hierarchical and systematic multi-objective and multi-criteria decision analysis method that combines qualitative and quantitative analysis. By applying the idea of system analysis, complex multi-objective and multi-criteria decision problems are transformed into simple quantitative decision problems. It has been widely used in the evaluation, prediction, system analysis, and other aspects. The main steps of AHP include three parts: establishing a hierarchical structure model, constructing a judgment matrix, and determining the weights of each main controlling factor^12^.

**Figure 7:**
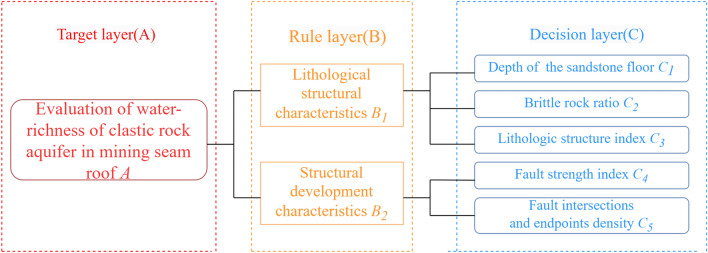
Hierarchical structure model.

1. Establishing a hierarchical structure model. The ultimate goal of this article was to evaluate the water-richness of the clastic rock aquifer on the roof of the mining coal seam, and used this as the target layer (A layer); the lithological structural characteristics and structural development characteristics reflected the water-richness of the aquifer, but their impact needed to be reflected through specific factors related to them as the rule layer of the model (B layer); the specific main controlling factors included the depth of the sandstone floor, brittle rock ratio, lithological structure index, fault strength index, fault intersections and endpoints density, which constituted the decision layer of the model (C layer). The hierarchical structure model was shown in Fig. 7.
2. Constructing a judgment matrix. Based on the varying degrees of influence of various main controlling factors on the evaluation of water-richness of sandstone aquifers, a quantitative analysis was conducted according to a certain scale to construct a judgment matrix. Collected the opinions of coal mine water prevention and control researchers and experts with rich on-site work experience in Huafeng mine field, and used the 1–9 scale method (Table 3) proposed by American operations researcher T.L. Satty^33^ to score the importance of the two major factors in the rule layer and the five main controlling factors in the decision layer, and established corresponding judgment matrices.
3. Consistency checking. Performed a consistency proportion test on each judgment matrix, and only after passing the consistency test can the weights obtained from the judgment matrix be accepted. First, the consistency index *CI* of the *n* order judgment matrix was calculated by the following formula:
  14 $$CI = \frac{{\lambda_{\max } - n}}{n - 1},$$
  where $\lambda_{\max }$ is the maximum eigenvalue of judgment matrix. *CI* = 0 indicates that the judgment matrix is completely consistent, *CI* < 0.1, the judgment matrix and single ranking of intra layer factors conform to logical consistency; if *CI* > 0.1, the assignment of factor weights within the judgment matrix needs to be adjusted.

**Table 3:** Saaty 1–9 rating scale.

Scale	Relative importance of two factors
1	Both factors are equally important
3	One factor is slightly more important than another
5	A certain factor is obviously more important
7	A certain factor is strongly more important
9	A certain factor is extremely more important
2, 4, 6, 8	The compromise value between the adjacent standards mentioned above

Then, the consistency ratio *CR* could be calculated by the following formula:

15 $$CR = \frac{CI}{{RI}},$$

where *RI* is the judgment matrix average random consistency indicator. *CR* < 0.1, the consistency of the judgment matrix is considered within the allowable range, and the weight vector calculation can be performed using the eigenvectors of the judgment matrix.

(4) Obtaining the weight of the main control factor. Using the square root method to calculate weights, firstly calculated the *n* power of the product of each row of the *n* order judgment matrix to obtain an *n* vector. The vector factor calculation formula was as follows:
  16 $$\overline{\omega }_{i} = \sqrt[n]{{\prod\limits_{j = 1}^{n} {a_{ij} } }},$$
  where $\overline{\omega }_{i}$ is the *I* element of an *n* dimensional vector; $a_{ij}$ is the scale value of the *I* row and the *j* column; *i*,*j* = 1, 2, …, *n*.

Normalizing the *n* dimensional vector mentioned above was the weight vector, which could obtain the weight:

17 $$\omega_{i} = \frac{{\overline{\omega }_{i} }}{{\sum\limits_{j = 1}^{n} {\overline{\omega }_{i} } }},$$

where $\omega_{i}$ is the weight value of the *I* main controlling factor.

### Calculating objective weights based on rough set theory improved by conditional entropy

Rough Set Theory is a mathematical method for dealing with fuzziness and uncertainty^34^. It can mine potential and valuable knowledge from a large amount of data, reducing the unnecessary workload caused by redundant knowledge in calculation and classification. When processing data, there is no need to provide prior information beyond the data, and the importance of each attribute can be determined. Currently, it is widely used in objective weight calculation. In the process of calculating attribute weights using rough set theory, there may be situations where the weight of a certain attribute is 0. The reason for this phenomenon is that Rough Set Theory only considers the importance of a single attribute to the entire attribute set, without considering the importance of the attribute itself and neglecting the practical significance of the attribute^35^. To solve this problem, the concept of Conditional Entropy is introduced to improve the method of calculating attribute weights using Rough Set Theory. The objective weight calculation steps for the main controlling factors of water-richness of clastic rocks on the roof of the mining coal seam were as follows.

(1) The knowledge system *S* for data processing related to water-richness evaluation was established as follows:

18 $$S = \left\langle {U,A,V,f} \right\rangle ,$$

where *U* is a set of objects, also known as a universe; $A = C \cup D, \, C \cap D = \emptyset$,* C* is the set of conditional attributes, *D* is the set of decision attributes; $V = U_{a \in A} V_{a}$ is a set of attribute values, *V*_*a*_ represents the range of attribute values for attribute *A*, that is, the range of values for attribute *a*; $f:U \times A \to V$ is an information function that specifies the attribute value of *x* for each object in *U*.

(2) Data standardization and classification. The selected controlling factors have different dimensions. To make the data more comparable and objective, the original data was standardized using the range standardization formula:

19 $$X_{i} = \frac{{X - X_{\min } }}{{X_{\max } - X_{\min } }},$$

where *X* is the original value of this indicator; *X*_max_, *X*_min_ are the maximum and minimum values in the original data of the indicator, respectively, *Xi* is the standardized result value of this indicator.

Divided the processed data into four levels based on intervals of [0, 0.25], (0.25, 0.5), (0.5, 0.75), and (0.75, 1), corresponding to the levels of small, medium, large, and extremely large mine water inflow.

(3) Reducing knowledge system data. In the decision table, if there were identical conditional attribute values and most of the decision attribute values were the same but differ, remove the few rows that caused the difference in the decision attributes and saved the rows with the same decision attribute values; If two rows with the same conditional attribute and different decision attribute values were encountered, these two rows could be deleted, as the sample had no specific significance for classification; If there were several rows in the policy table with consistent values for conditional attributes or decision attributes, then kept one of them^36^.

(4) Using Conditional Entropy to improve the Rough Set method for calculating attribute weights.

a. Calculating the conditional entropy of decision attributes. In decision information table $S = \left\langle {U,C,D,V,f} \right\rangle$, the conditional entropy of decision attribute set $D\left( {U/D = \left\{ {D_{1} ,D_{2} , \cdots ,D_{k} } \right\}} \right)$ relative to conditional attribute set $C\left( {U/C = \left\{ {C_{1} ,C_{2} , \cdots ,C_{m} } \right\}} \right)$ could be expressed as:

20 $$I(D|C) = \sum\limits_{i = 1}^{m} {\frac{{|C_{i} |^{2} }}{{|U|^{2} }}} \sum\limits_{j = 1}^{k} {\frac{{|D_{j} \cap C_{i} |}}{{|C_{i} |}}} \, \times \left[ {1 - \frac{{|D_{j} \cap C_{i} |}}{{|C_{i} |}}} \right],$$

b. Calculating the importance of the condition attribute *C*_*i*_. In the decision information table, $\forall Ci \in C$, the importance of the conditional attribute *C*_*i*_ could be expressed as:

21 $$New \, Sig\left( {C_{i} } \right) = I(D|C - C_{i} ) - I(D|C),$$

where $New \, Sig\left( {C_{i} } \right)$ represents the degree of change in the attribute set after removing the conditional attribute *C*_*i*_, indicating the importance of the conditional attribute *C*_*i*_ relative to the entire conditional attribute set.

c. Calculating attribute weights. By comprehensively considering the above two aspects and standardizing them, the weights of each conditional attribute (factor) could be obtained:

22 $$W\left( {C_{i} } \right) = \frac{{New \, Sig\left( {C_{i} } \right) + I(D|C_{i} )}}{{\sum\limits_{i = 1}^{m} {\left\{ {New \, Sig\left( {C_{i} } \right) + I(D|C_{i} )} \right\}} }},$$

where $I(D|C_{i} )$ indicates the importance of the conditional attribute *C*_*i*_ itself in the system.

According to the above steps, the conditional entropy, importance, and weights of each main controlling factor could be obtained.

### Calculating comprehensive weights based on the minimum deviation method

After calculating the subjective and objective weights using AHP and improved rough set theory, to balance the subjectivity and objectivity of the weight indicators and complement each other’s strengths and weaknesses, a combination weighting method based on minimum deviation was adopted to fuse the subjective and objective weights, reduced the errors caused by a single weighting method, and obtained a more scientific and reasonable comprehensive weight^37,38^.

Assuming that decision-makers use a total of *q* methods to determine indicator weights, including subjective weighting method *l* and objective weighting method *q*-*l*, the weight vector is:

23 $$u_{k} = \left( {u_{k1} ,u_{k2} , \cdots ,u_{km} } \right)^{T} \, k = 1,2, \cdots ,q$$

24 $$\sum\limits_{i = 1}^{m} {u_{ki} = 1} .$$

In order to comprehensively consider the subjective opinions of decision-makers and the objectivity of decision-making, a deviation function is introduced to minimize the weight deviation obtained by various weighting methods, and ultimately obtain the weight vector $w = \left\{ {w_{1} ,w_{2} , \cdots ,w_{m} } \right\}^{T}$. The specific steps are as follows.

(1) Constructing a single objective optimization model:

25 $$\min J = \sum\limits_{k = 1}^{l} {\sum\limits_{j = 1}^{n} {a_{k} f_{j} \left( {u_{k} } \right)} } + \sum\limits_{k = l + 1}^{q} {\sum\limits_{j = 1}^{n} {a_{k} g_{j} \left( {u_{k} } \right)} } ,$$

26 $$s.t. \, \sum\limits_{i = 1}^{m} {\omega_{i} = 1} ; \, \omega_{i} \ge 0$$

where *a*_*k*_ is the weight coefficient corresponding to various weighting methods; *a*_*j*_ is the weight corresponding to the *j* weighting method;$f_{j} \left( {u_{k} } \right)$, $g_{j} \left( {u_{k} } \right)$ is the deviation function of subjective weighting method and objective weighting method, respectively.

In order to fully utilize the weight information determined by various weighting methods, the weight deviation of each weighting method should be smaller. Therefore, the constructed model is:

27 $$\min J = \mathop \sum \limits_{j = 1}^{n} \mathop \sum \limits_{k = 1}^{q} \mathop \sum \limits_{i = 1}^{m} \left( {a_{k} u_{ki} - a_{j} u_{ij} } \right)^{2} ,$$

28 $$s.t. \, \sum\limits_{k = 1}^{q} {a_{k} } = 1; \, a_{k} \ge 0; \, k \in [1,q]$$

(2) Constructing the corresponding Lagrange function:

29 $$L\left( {a,\lambda } \right) = \sum\limits_{j = 1}^{n} {\sum\limits_{k = 1}^{q} {\sum\limits_{i = 1}^{m} {\left( {a_{k} u_{ki} - a_{k} u_{ij} } \right)} } }^{2} + \lambda \left( {\sum\limits_{k = 1}^{q} {a_{k} - 1} } \right)$$

where λ is an introduced parameter.

According to the necessary conditions for the existence of extreme values, there are:

30 $$\left\{ {\begin{array}{*{20}c} {\frac{\partial L}{{\partial a_{k} }} = qa_{k} \sum\limits_{i = 1}^{m} {u_{ki}^{2} } - \alpha_{1} \sum\limits_{i = 1}^{m} {u_{1i} } u_{ki} - \alpha_{2} \sum\limits_{i = 1}^{m} {u_{2i} } u_{ki} - \cdots - \alpha_{q} \sum\limits_{i = 1}^{m} {u_{qi} } u_{ki} + \frac{\lambda }{2} = 0,} \\ {\frac{\partial L}{{\partial \lambda }} = \sum\limits_{k = 1}^{q} {a_{k} } - 1 = 0,} \\ \end{array} } \right.$$

where *L* is the constructed Lagrange function.

According to Kramer's law, the coefficient determinant of the linear equation system is not 0, so the equation system has a unique solution, which is the corresponding weight coefficients of various weighting methods. By weighting them with the indicator weights of the corresponding methods, the final weight value can be obtained.

(3) Obtaining a system of comprehensive weighting equations. By substituting the weight values obtained from the two weighting methods into the Lagrange function mentioned above, the equation system for obtaining the comprehensive weighting can be obtained:

31 $$\left\{ {\begin{array}{*{20}c} {\left( {\sum\limits_{i = 1}^{n} {u_{1i}^{2} } } \right)\alpha_{1} - \left( {\sum\limits_{i = 1}^{n} {u_{2i} u_{1i} } } \right)\alpha_{2} + \frac{\lambda }{2} = 0,} \\ \begin{gathered} \hfill \\ - \left( {\sum\limits_{i = 1}^{n} {u_{1i} u_{2i} } } \right)\alpha_{1} + \left( {\sum\limits_{i = 1}^{n} {u_{2i}^{2} } } \right)\alpha_{2} + \frac{\lambda }{2} = 0, \hfill \\ \end{gathered} \\ \begin{gathered} \hfill \\ \alpha_{1} + \alpha_{2} = 1, \hfill \\ \end{gathered} \\ \end{array} } \right.$$

(4) Obtaining comprehensive weights. Substituting the obtained subjective and objective weight, $\alpha * = \left( {\alpha_{1} , \, \alpha_{2} } \right)$ could be obtained. The comprehensive weight calculation of the main controlling factors for water-richness evaluation in this article was as follows:

32 $$\omega_{i} = \alpha_{1} u_{1i} + \alpha_{2} u_{2i} ,$$

where $\alpha_{1}$ is the weight coefficient of subjective weight; $\alpha_{2}$ is the weight coefficient of objective weight; $u_{1i}$ is the subjective weight; $u_{2i}$ is the objective weight.

### Constructing a water-richness classification model based on unascertained measurement theory

The unknown measurement theory was introduced into the evaluation of the water-richness of the coal seam roof water-bearing layer. This theory satisfies the criteria of non-negativity, additivity, normalization, and temporality, and applies the principle of confidence. The advantage of the comprehensive evaluation model based on the measurement mathematics is that no useful information will be lost when making judgments, and the use of the provided confidence criteria will not result in unclear or unreasonable classifications as in the maximum membership principle, especially for the problem of ordered partition classes, the classification degree is more accurate and detailed^39^. The steps for constructing the evaluation model were as follows.

(1) Classifying evaluation indicators.

When evaluating the target with an unascertained measure set, the key was constructing a reasonable unascertained measure function, and the first step was to establish a risk assessment level. On the basis of thorough research and analysis of the hydrogeological characteristics of the Huafeng mine field, a water-richness evaluation index for the study area was established through K-means clustering analysis and combined with the opinions of coal mine water prevention and control researchers, as shown in Table 4. Divided the water-richness in the study area into four levels, namely strong water-richness (C4), relatively strong water-richness (C3), medium water-richness (C2), and relatively weak water-richness (C1).

**Table 4:** Evaluation indicators and grading standards.

Evaluation indicators	Strong water-rich (C4)	Relatively strong water-rich (C3)	Medium water-rich (C2)	Relatively weak water-rich (C1)
Depth of the sandstone floor (m)	> 1200	(800, 1200)	(500, 800)	< 500
Brittle rock ratio	> 0.9	(0.7, 0.9)	(0.5, 0.7)	< 0.5
Lithological structure index	> 50	(35, 50)	(25, 35)	< 25
Fault strength index	> 0.09	(0.052, 0.09)	(0.014, 0.052)	< 0.014
Fault intersections and endpoints density	> 100	(60, 100)	(20, 60)	< 20

(2) Constructing a single indicator measurement function.

Assuming there are *n* evaluation units in the aquifer to be evaluated, the space vector *Q* = {Q_1_, Q_2_, Q_3_, …, Q_*n*_} can be used to represent it. For each unit Q_*i*_ (*I* = 1, 2, …, *n*) to be evaluated, there are *m* evaluation indicators, namely *X* = {X_1_, X_2_, X_3_, …, X_*m*_}. If X_*ij*_ represents the quantitative value of the *j* evaluation indicator of the evaluation unit Q_*i*_, then the quantitative value of the evaluation indicator Q_*i*_ of the evaluation unit *Q*_*i*_ = {X_*i*1_, X_*i*2_, X_*i*3_, …, X_*im*_}. If Q*i* has *s* levels of evaluation, then the level space *R* = {*C*_1_, *C*_2_, *C*_3_, …, *C*_s_ }. Let *C*_*k*_ (*k* = 1, 2, …, s) be the *k* level evaluation, if it satisfies *C*_1_ > *C*_2_ > *C*_3_ > … > *C*_*s*_, then {*C*_1_, C_2_, C_3_, …, C_s_} is called an ordered partition class of the level space *R*.

Let $\alpha_{ijk} = \alpha \left( {X_{ij} \in C_{k} } \right)$ represent the degree to which the quantified value of the evaluation index belongs to the *k* evaluation level *C*_*k*_. If α satisfies $0 \le \alpha \left( {X_{ij} \in C_{k} } \right) \le 1, \, \alpha \left( {X_{ij} \in R} \right) = 1$,$\alpha \left( {X_{ij} \in C_{l} } \right) = \sum\limits_{l = 1}^{k} {\alpha \left( {X_{ij} \in C_{l} } \right)}$, it is called an uncertain measure, abbreviated as a measure. A matrix $\left( {\alpha_{ijk} } \right)_{m \times s}$ is called a single-index measure evaluation matrix, that is:

33 $$\left( {\alpha_{ijk} } \right)_{m \times s} = \left[ {\begin{array}{*{20}c} {\begin{array}{*{20}c} {\alpha_{i11} } & {\alpha_{i12} } \\ \end{array} } & {\begin{array}{*{20}c} \cdots & {\alpha_{i1s} } \\ \end{array} } \\ {\begin{array}{*{20}c} {\begin{array}{*{20}c} {\alpha_{i21} } & {\alpha_{i22} } \\ \end{array} } \\ \vdots \\ {\begin{array}{*{20}c} {\alpha_{im1} } & {\alpha_{im2} } \\ \end{array} } \\ \end{array} } & {\begin{array}{*{20}c} {\begin{array}{*{20}c} \cdots & {\alpha_{i2s} } \\ \end{array} } \\ {\begin{array}{*{20}c} \ddots & \vdots \\ \end{array} } \\ {\begin{array}{*{20}c} \cdots & {\alpha_{ims} } \\ \end{array} } \\ \end{array} } \\ \end{array} } \right].$$

**Figure 8:**
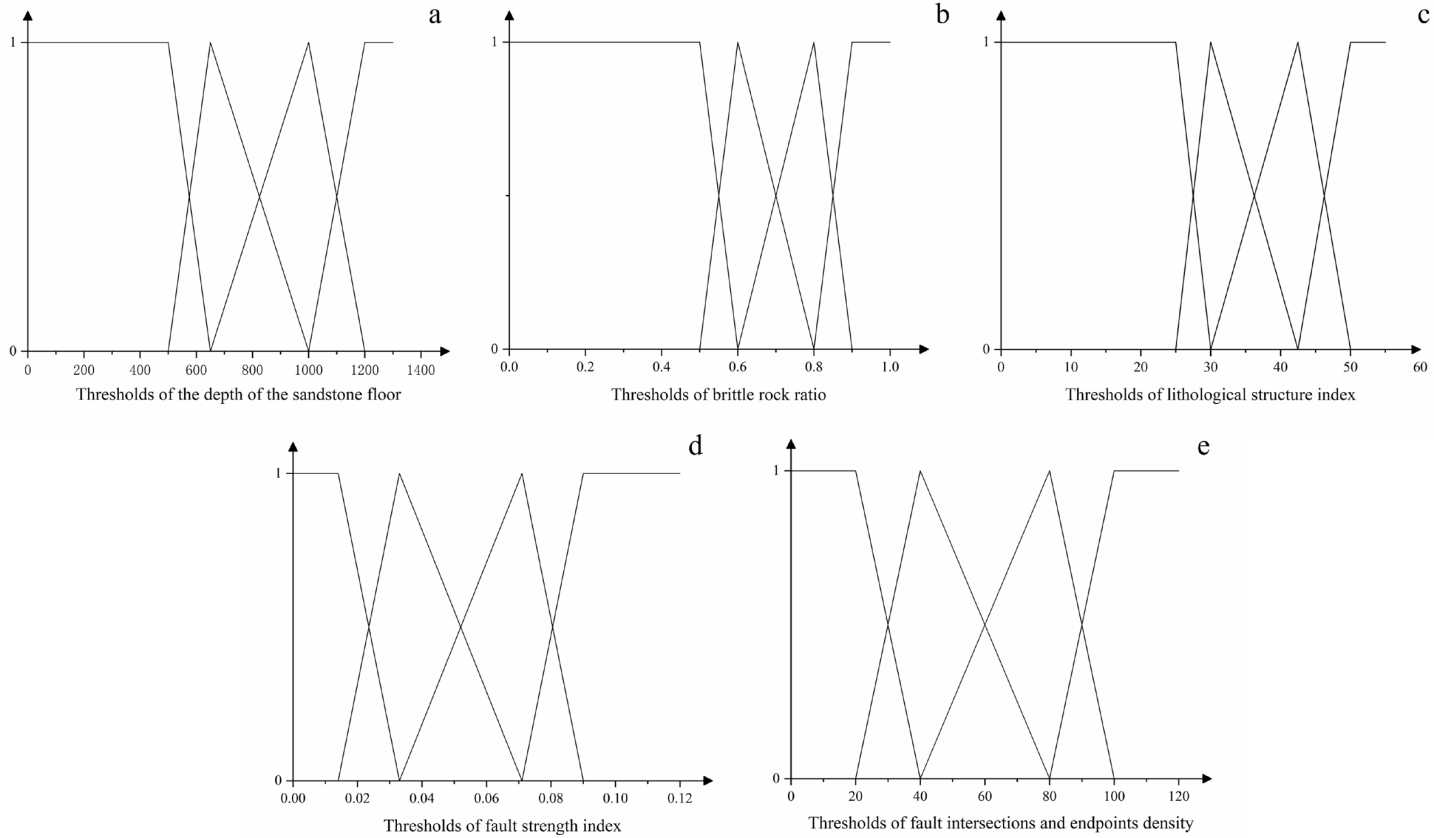
The linear measure function images.

According to the controlling factors and the characteristics of actual mining, this paper performed linear interpolation within the interval, that was, the idea of piecewise interpolation was used to insert linear points in each graded interval of the evaluation index. Based on the inserted points, a linear measure function was constructed as shown in Fig. 8.

(3) Constructing a comprehensive measure of multiple indicators based on indicator weights.

Let $\alpha_{ik} = \alpha \left( {Q_{i} \in C_{k} } \right)$ represent the degree to which the evaluation unit Q_*i*_ belongs to the *k* evaluation level *C*_*k*_. Then, the multi-index measure $\alpha_{ik} = \sum\limits_{j = 1}^{m} {\omega_{j} \alpha_{ijk} }$ can be represented by a matrix as follows:

34 $$\left( {\alpha_{ik} } \right)_{n \times s} = \left[ {\begin{array}{*{20}c} {\begin{array}{*{20}c} {\alpha_{11} } & {\alpha_{12} } \\ \end{array} } & {\begin{array}{*{20}c} \cdots & {\alpha_{1s} } \\ \end{array} } \\ {\begin{array}{*{20}c} {\begin{array}{*{20}c} {\alpha_{21} } & {\alpha_{22} } \\ \end{array} } \\ \vdots \\ {\begin{array}{*{20}c} {\alpha_{n1} } & {\alpha_{n2} } \\ \end{array} } \\ \end{array} } & {\begin{array}{*{20}c} {\begin{array}{*{20}c} \cdots & {\alpha_{2s} } \\ \end{array} } \\ {\begin{array}{*{20}c} \ddots & \vdots \\ \end{array} } \\ {\begin{array}{*{20}c} \cdots & {\alpha_{ns} } \\ \end{array} } \\ \end{array} } \\ \end{array} } \right].$$

(4) Grading through confidence recognition criteria.

To obtain the final evaluation result of the evaluation unit, a "confidence degree" identification criterion is adopted for the grading evaluation of aquifer water abundance^40^. If *R* = (*c*_1_ > *c*_2_ > *c*_3_ … > *c*_z_), the evaluation space *R* is considered ordered. The confidence degree λ is introduced, where λ ≥ 0.5, and if $k_{0} = \min \left\{ {k:\sum\limits_{l = 1}^{k} {\alpha il \ge \lambda , \, \left( {k = 1,2, \cdots ,s} \right)} } \right\}$, it can be regarded that the evaluation unit Q*i* belongs to the *k*_0_ evaluation level *C*_*k*0_.

## Results and analysis

### Prediction of the development height of water-conducting fracture zones

1. *Empirical formulas* The lithology of the roof strata of the 4th coal seam in the Huafeng mine field was analyzed, and different empirical formulas were used to calculate the development height of the water-conducting fractured zone based on the different roof lithologies. The predicted values using the empirical formula method were plotted using Golden Software Surfer software, as shown in Fig. 10a.
2. *The GRNN model* A total of 63 measured cases were collected and organized, forming a 63 × 5-dimensional raw data matrix (Table 5) (see the Supplementary Information 1). The selected four influencing factors were used as input parameters for the GRNN model, and the predicted values of the development height of the water-conducting fractured zone were used as output parameters. Since the number of layer parameters in the GRNN neural network model is equal to the number of input layers, and the number of summation layer parameters is one more than the output layer, the initial network structure of the GRNN neural network was designed as 4:4:2:1. Samples numbered 1 to 58 in the table were used as training samples, while samples numbered 59 to 63 were used as testing samples. The GRNN neural network model was trained using Matlab software, and the smoothing factor σ was adjusted to achieve the best fit of the training results. Finally, a GRNN neural network with a fitting degree of 0.9023 was obtained.

**Table 5:** Measured sample data of the height of the water-conducting fractured zone.

Sample number	*m* (m)	*b*	*l* (m)	*s* (m)	*H* (m)	Sample number	*m* (m)	*b*	*l* (m)	*s* (m)	*H* (m)
1	8.7	0.45	198	418	83	33	4.5	0.45	135	370	57.5
2	8.6	0.38	170	357	66.5	34	3	0.52	209	420.5	52.01
3	8.6	0.41	190	367	61.8	35	4.5	0.52	147	499	67.88
4	8.7	0.62	153	434	71	36	5	0.5	174	489.43	73.28
5	3	0.94	227	367	32.5	37	5	0.35	200	520	58.46
6	9	0.51	220	590	76	38	5.62	0.79	224.26	462	95.07
7	7.6	0.62	116	463	86.4	39	3.6	0.25	301	288	44.98
8	3	0.23	186	649.1	42.99	40	5.1	0.82	78	266.3	51.3
9	5	0.81	122	320	67.7	41	8	0.53	120	272	62
10	4.8	0.36	175	485	62.5	42	7.69	0.51	240	207	62.31
11	4.6	0.5	170	86.1	53.9	43	4.5	0.47	160	489	54.79
12	3.8	0.65	168	270	54.6	44	4.5	0.53	132	472.5	57.45
13	7	0.52	168	433	72.97	45	2.03	0.95	69	89	45.86
14	7.4	0.55	160	331	64.25	46	3.4	0.26	120	424.42	45.1
15	5.3	0.24	145.7	312	44.2	47	3.9	0.28	209	475.2	49.05
16	5.7	0.63	177.9	283	51.4	48	5.8	0.45	186	557.25	65.25
17	8	0.55	170	450	86.8	49	4.5	0.55	175	387.5	48.9
18	2.94	0.85	180.4	568.4	57	50	3.65	0.63	132	476.4	55
19	2.95	0.74	206.1	516	54.5	51	5.7	0.63	177.9	283.9	54.79
20	7.5	0.19	222	665	53.7	52	4	0.52	135	490	45
21	4.5	0.55	175	387.5	58.5	53	3.4	0.46	136	434.4	45.1
22	8	0.53	198	781.1	97.7	54	4	0.07	195	445.4	38.81
23	9.5	0.65	123	450	78	55	4.8	0.47	150	499.92	54
24	13.425	0.7	123	490	130.78	56	2	0.53	105	351.3	36.99
25	4.7	0.39	297	368	56	57	7.5	0.47	174	367	75.5
26	5.8	0.34	178	570	65.25	58	4.6	0.43	120	427.3	56.6
27	3.4	0.46	120	434	45	59	7.53	0.38	170	357	61.9
28	3	0.35	145	434.1	47.55	60	7.52	0.41	190	367	61.77
29	3.4	0.36	120	441.97	48.9	61	2.8	0.68	156	269	50.34
30	3.5	0.68	145	452.7	75.8	62	6.1	0.37	170	475	64.6
31	3.65	0.65	132	568.6	60.14	63	3.7	0.71	70	420	56.8
32	3.85	0.54	209	478.3	52.15						

The coal seam thickness, proportion coefficient of hard rock lithology, dip length of the workface, and mining depth of the 4th coal seam in the Huafeng mine field were used as input parameters, and the developed GRNN model was utilized to predict the development height of the water-conducting fractured zone. The results were shown in Fig. 10b.

**Figure 9:**
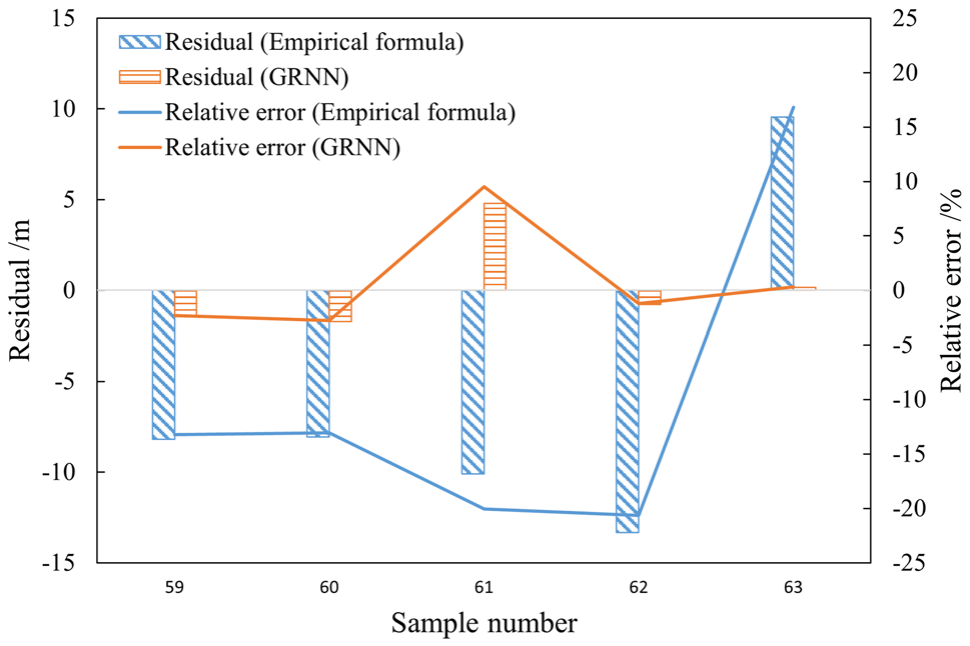
Residual and relative error of different methods.

The predicted values of water-conducting fractured zone development height for the test samples obtained from the GRNN neural network method and empirical formula method were compared. The relative error of the two methods was shown in Fig. 9, and the average relative errors were 3.22% and 16.75%, respectively. The prediction accuracy of the GRNN neural network method was significantly higher than that of the empirical formula method.

**Figure 10:**
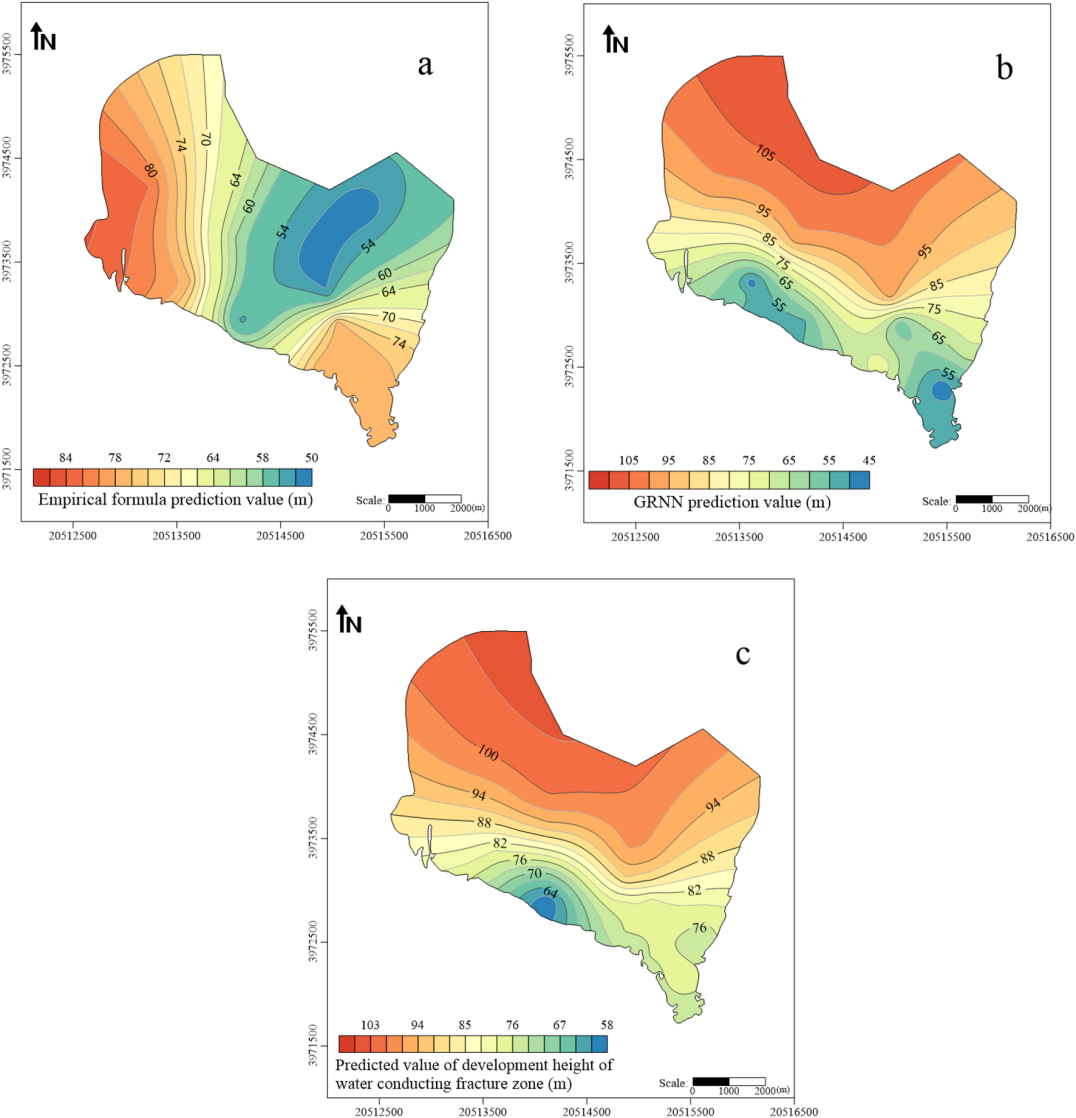
Predicted value of development height of water-conducting fracture zone.

The development height of the water-conducting fractured zone in the coal seam roof was calculated using the two aforementioned methods. The GRNN neural network method exhibited higher accuracy; however, prioritizing safety, the larger prediction value of the two methods was chosen as the final prediction value. A thematic map illustrating the predicted values of the water-conducting fractured zone development height was generated using Golden Software Surfer, as shown in Fig. 10c. The development trend of the water-conducting fractured zone in the roof of the 4th coal seam followed a relatively regular pattern, gradually increasing in height from south to north. The lowest predicted value for the development height of the water-conducting fractured zone was 58 m, while the highest reached up to 106 m.

### Calculation of the weight values of the main controlling factors for water-richness

1. Subjective weight. We invited a total of five researchers in coal mine water prevention and control and experts with rich on-site work experience in Huafeng mine field to evaluate and score the relative importance of the main controlling factors for water-richness of the clastic rock aquifer of the mining coal seam roof (Table 6), and constructed a judgment matrix, as shown in Tables 7, 8 and 9. Conducted a consistency check on each judgment matrix, and the test results were shown in the table. The consistency ratio *CR* was less than 0.1 (Table 10), which met the consistency requirements of the judgment matrix. The weight of each main controlling factor was shown in Table 11.
2. Objective weight. According to Rough Set Theory, by removing redundant parts from the data and combining the concept of Conditional Entropy, following the previously mentioned calculation steps of attribute weights, the objective weights of the main controlling factors for water abundance were obtained as shown in Table 12.
3. Combination weights. After obtaining the subjective and objective weights of each main controlling factor, the weight coefficients of the subjective and objective weights could be obtained by substituting them into Eq. (31), ${\alpha }_{1}$, ${\alpha }_{2}$ were 0.495 and 0.505 respectively (The table used for the calculation is shown in Supplementary Information 2). Therefore, the combination weighting model in this article was $\omega_{i} = 0.495u_{1i} + 0.505u_{2i}$.

**Table 6:** Assessment of the main controlling factors.

Researchers	B1&B2	C1&C2	C1&C3	C2&C3	C4&C5
1	2	3	1	1	3
2	3	2	1	1	3
3	2	2	1	1	1
4	1	1	1	1	1
5	2	2	1	1	2

**Table 7:** Judgment matrix ${\varvec{A}}\sim {{\varvec{B}}}_{{\varvec{i}}}({\varvec{i}}=1\sim 2)$.

Evaluation of aquifer abundance *A*	Lithological structural characteristics	Structural development characteristics	*W*
Lithological structural characteristics *B*_*1*_	1	2	0.6667
Structural development characteristics *B*_*2*_	0.5	1	0.3333

**Table 8:** Judgment matrix ${{\varvec{B}}}_{1}\sim {{\varvec{C}}}_{{\varvec{i}}}({\varvec{i}}=1\sim 3)$.

Lithological structural characteristics *B*_*1*_	Depth of the sandstone floor	Brittle rock ratio	Lithological structure index	*W*
Depth of the sandstone floor *C*_*1*_	1	0.5	1	0.2611
Brittle rock ratio *C*_*2*_	2	1	1	0.4111
Lithological structure index *C*_*3*_	1	1	1	0.3278

**Table 9:** Judgment matrix ${{\varvec{B}}}_{2}\sim {{\varvec{C}}}_{{\varvec{i}}}({\varvec{i}}=4\sim 5)$.

Structural development characteristics *B*_*2*_	Fault strength index	Fault intersections and endpoints density	*W*
Fault strength index *C*_*4*_	1	2	0.6667
Fault intersections and endpoints density *C*_*5*_	0.5	1	0.3333

**Table 10:** Consistency check table for judgment matrix.

Matrix	${\lambda }_{max}$	*CI*	*CR*
$A\sim {B}_{i}(i=1\sim 2)$	2.0000	0	0.0000
${B}_{1}\sim {C}_{i}(i=1\sim 3)$	3.0537	0.0269	0.0516
${B}_{2}\sim {C}_{i}(i=4\sim 5)$	2.0000	0	0.0000

**Table 11:** Subjective weight of main controlling factors.

	Depth of the sandstone floor	Brittle rock ratio	Lithological structure index	Fault strength index	Fault intersections and endpoints density
*W*	0.1741	0.2741	0.2185	0.2222	0.1111

**Table 12:** Objective weight of main controlling factors.

	Depth of the sandstone floor	Brittle rock ratio	Lithological structure index	Fault strength index	Fault intersections and endpoints density
$I\left(D|{C}_{i}\right)$	0.2116	0.3555	0.2787	0.3020	0.2192
$I\left(D|C-{C}_{i}\right)$	0.0000	0.0000	0.0000	0.0000	0.0000
$New Sig\left({C}_{i}\right)$	0.0000	0.0000	0.0000	0.0000	0.0000
*W*	0.1548	0.2600	0.2039	0.2209	0.1604

**Figure 11:**
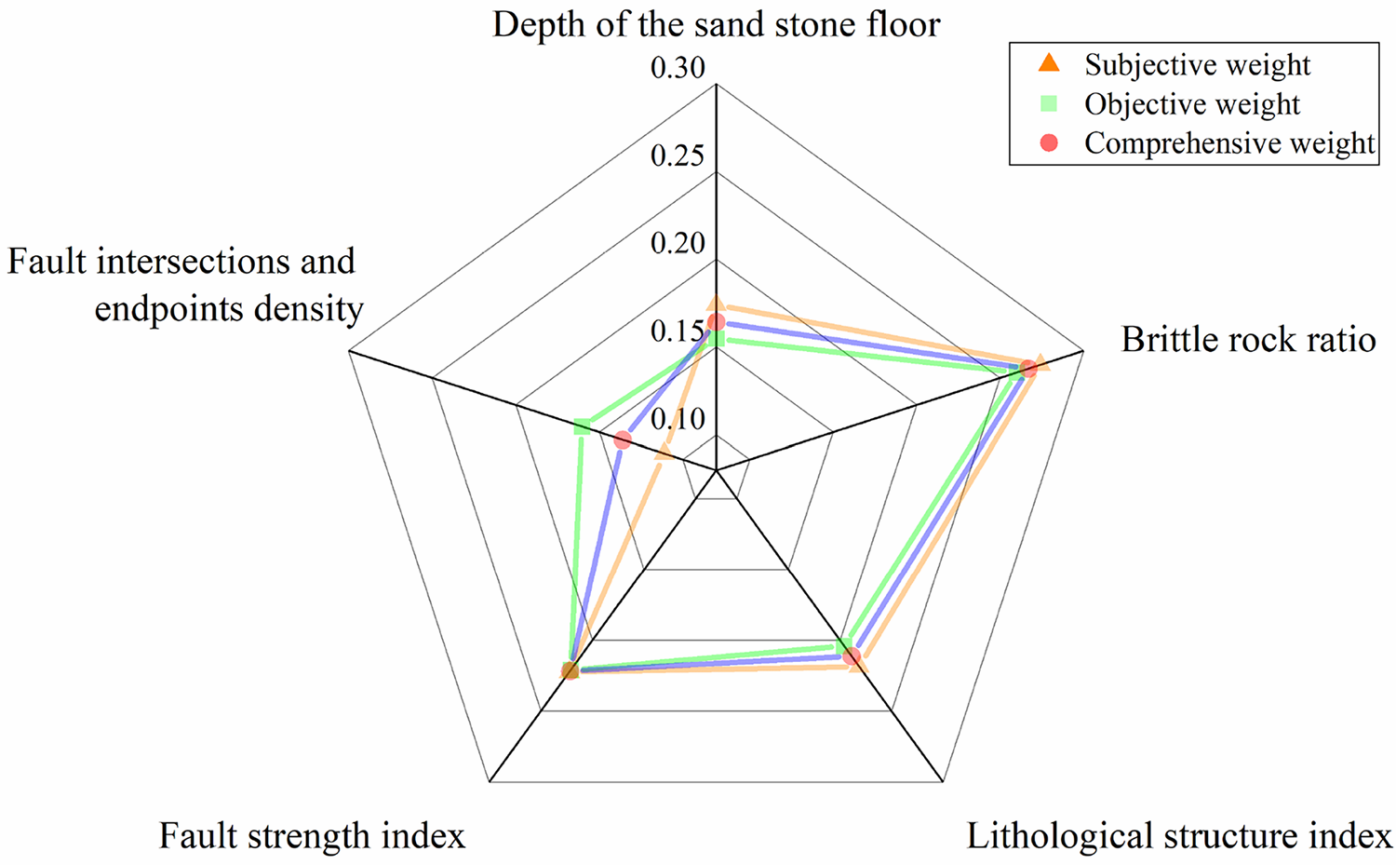
Weighted radar chart.

From this, the final comprehensive weight of each main control factor could be obtained: the depth of the sandstone floor was 0.1644, the brittle rock ratio was 0.267, the lithological structure index was 0.2111, the fault strength index was 0.2215, and the fault intersections and endpoints density was 0.136. Draw a weighted radar chart to visualize the subjective, objective, and comprehensive weights, as shown in Fig. 11.

### Constructing the water-richness evaluation model

The mine field was divided into evaluation units with a size of 250 m $\times$ 250 m. Based on the previously collected and organized data on the controlling factors of water-richness evaluation, the central coordinates of each unit were used to perform Kriging interpolation using Golden Software Surfer. The evaluation units were used as the basis for constructing the water-richness zoning evaluation of the clastic aquifer in the 4th coal seam roof of the Huafeng mine field. Due to space limitations, it was not possible to list all the data for each evaluation unit. Only one evaluation unit, DY59, in the central part of the study area, would be used as an example to illustrate the water-richness rating based on the theory of uncertain measurement and the confidence criterion. The calculation method was as follows.

(1) Calculating the single indicator measure evaluation matrix.

Collected and organized the main controlling factor values of evaluation unit DY59, as shown in Table 13.

**Table 13:** Measured values of the main controlling factors of DY59.

Name	Depth of the sandstone floor	Brittle rock ratio	Lithological structure index	Fault strength index	Fault intersections and endpoints density
DY59	1001.07	0.73	43.81	0.0000	0.0000

By substituting the values of each main controlling factor into the linear measure function constructed, the single indicator measure evaluation matrix could be obtained as follows:

$$\alpha_{ijk} = \left[ {\begin{array}{*{20}c} {\begin{array}{*{20}c} {} & {} \\ {} & { \, 0.331} \\ \end{array} } & {\begin{array}{*{20}c} {\begin{array}{*{20}c} {} & 1 & 1 \\ \end{array} } \\ {\begin{array}{*{20}c} {} & {} & {} \\ \end{array} } \\ \end{array} } \\ {\begin{array}{*{20}c} {0.995} & {0.669} \\ {0.005} & {} \\ \end{array} } & {\begin{array}{*{20}c} {\begin{array}{*{20}c} {0.826} & {} & {} \\ \end{array} } \\ {\begin{array}{*{20}c} {0.174} & {} & {} \\ \end{array} } \\ \end{array} } \\ \end{array} } \right].$$

(2) Calculating the multi-indicator comprehensive measure matrix.

In 3.3.3 of this article, the comprehensive weight of the main controlling factors for water-richness evaluation was obtained, and the comprehensive weight vector was $\omega = \left\{ {0.1644,0.267,0.2111,0.2215,0.136} \right\}$, According to the equation $\alpha_{ik} = \sum\limits_{j = 1}^{m} {\omega_{j} \alpha_{ijk} }$, the evaluation vector $\alpha_{ik} = \left\{ {0.358,0.088,0.517,0.038} \right\}$ of the multi-indicator comprehensive measure of DY59 could be obtained.

(3) The confidence criterion determines the evaluation level.

With a confidence level of λ = 0.5, the water-richness level of the DY59 evaluation unit was classified. When *k* was sorted from small to large, *k*_0_ = C_1_ + C_2_ + C_3_ = 0.358 + 0.088 + 0.517 = 0 0.962 > λ (0.5). When *k* is sorted from large to small, *k*_0_ = C_4_ + C_3_ = 0.555 > λ (0.5). Therefore, the water-richness level of the DY59 evaluation unit is relatively strong (C3).

**Figure 12:**
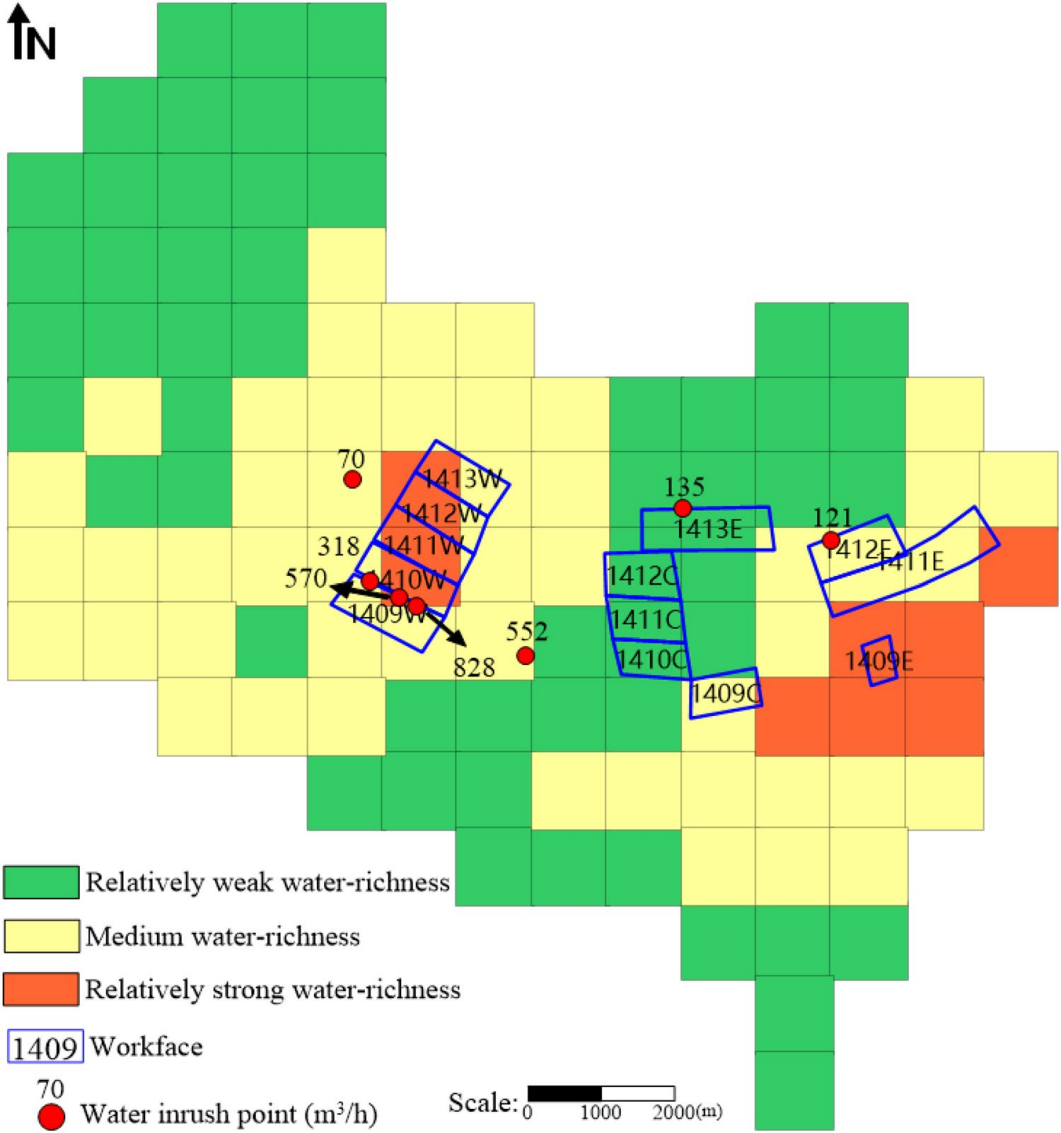
Water-richness zoning map of clastic rock aquifers on the roof of coal seam 4 in Huafeng mine field.

Through the aforementioned calculation steps, based on the theory of uncertain measurement and the confidence criterion, the water-richness evaluation level of all evaluation units within the study area could be computed, thus obtaining the water-richness zoning results of the aquifer in the mine field (The modeling process is demonstrated in Supplementary Information 3). The water-richness zoning map of the clastic aquifer in the 4th coal seam roof of the Huafeng mine field was created using Golden Software Surfer, as shown in Fig. 12. The water-richness in the study area was categorized into three levels: relatively weak water-richness (C1), medium water-richness (C2), and relatively strong water-richness (C3), with an overall low water-richness. The relatively weak water-richness zone occupied most of the study area, while the medium water-richness zone was distributed in the southwestern, central, and eastern parts of the study area. The relatively strong water-richness zone was present in small portions of the central and eastern parts of the study area.

Combined with the water inflow data and water inrush point records provided by the research area, the actual results were compared with the water abundance zoning model in this paper, as shown in Table 14.

**Table 14:** Validation between prediction results and actual data.

No. of water inrush point/workface		Actual yield (m^3^/h)	Prediction results	Actual yield description	Prediction results and field results comparison
No. of water inrush point	2003–3	318	Medium	Medium	Agree
	2004–1	570	Relatively strong	Medium	Disagree
	2004–2	858	Relatively strong	Large	Agree
	2004–3	552	Medium	Medium	Agree
	2016–10	121	Medium	Medium	Agree
	2017–3	70	Medium	Medium	Agree
	2020–1	135	Relatively weak	Medium	Disagree
No. of workface	1409W	744–816	Medium-relatively strong	Large	Agree in general
	1409C	300–360	Medium	Medium-large	Agree in general
	1409E	240–480	Relatively strong	Medium-large	Agree in general
	1410W	492–504	Medium-relatively strong	Large	Agree in general
	1410C	258	Relatively weak	Medium	Disagree
	1411W	126–156	Medium-relatively strong	Medium	Agree in general
	1411C	90–144	Relatively weak	Small-medium	Agree in general
	1411E	124–153	Medium	Medium	Agree
	1412W	120–180	Medium-relatively strong	Medium	Agree in general
	1412C	91–138	Relatively weak	Small-medium	Agree in general
	1412E	80–121	Medium	Small-medium	Agree in general
	1413W	30–60	Medium-relatively strong	Small	Disagree
	1413E	22–82	Relatively weak	Small	Agree

Taking the water inrush data as an example, out of 7 water inrush points, 2 of them did not match the model zoning, resulting in an accuracy rate of 71.43% for the model. Taking the water inflow data from workfaces as an example, out of 13 workfaces, 2 of them did not match the model zoning for water inflow, while 9 of them matched the model zoning to a certain extent, resulting in an accuracy rate of 84.62% for the model. Overall, the accuracy rate of the water-richness zoning model in this study could reach 80%.

## Conclusions

1. The method combining the GRNN neural network with empirical formulas was employed to calculate the predicted values of the development height of the water-conducting fracture zone above the mined coal seam. The predicted values for the development height of the water-conducting fracture zone in the 4th coal seam roof of the Huafeng mine field ranged from 58 to 106 m. This range was determined to assess the vertical extent of water-richness evaluation. Through quantitative analysis, the degree of water hazard threat to the mined coal seam was determined, thereby improving the accuracy of evaluating the water-richness of the clastic aquifer in the coal seam roof.
2. Depth of the sandstone floor, brittle rock ratio, lithological structure index, fault strength index, fault intersections and endpoints density were selected as the main controlling factors for water-richness in the study area, breaking away from the previous reliance on the unit water inflow factor. The method of the minimum deviation was introduced to optimize the combination of subjective weights obtained from the AHP and objective weights derived from the rough set theory improved by conditional entropy. This resulted in a combined weight that possesses the advantages of both subjective and objective weights. The combination weights for each main controlling factor are 0.1644, 0.267, 0.2111, 0.2215, and 0.136 in sequence. This significantly improves the reliability and accuracy of the weights, making the evaluation results more scientifically sound.
3. The theory of uncertain measurement was applied to the construction of a water-richness evaluation model by incorporating confidence identification criteria. The model was used to classify the water-richness of the clastic rock aquifer in the 4th coal seam roof of the Huafeng mine field. It was divided into relatively strong water-richness zones, medium water-richness zones, and relatively weak water-richness zones. By comparing the records of water inflow from workfaces and water inrush points within the study area, a high degree of agreement was observed, reaching 80% accuracy. This validates the feasibility and accuracy of the proposed method in this study.

### Supplementary Information

Supplementary Information 1.

Supplementary Information 2.

Supplementary Information 3.

## Data Availability

The datasets used and/or analyzedduring the current study are available from the corresponding author upon reasonable request.
